# Aortic coarctation augments changes in thoracic aortic hemodynamics in pediatric and young adult patients with bicuspid aortic valve

**DOI:** 10.1186/1532-429X-15-S1-P300

**Published:** 2013-01-30

**Authors:** Bradley D Allen, Alex J Barker, Maya Gabbour, Michael Markl, Cynthia Rigsby, Joshua D Robinson

**Affiliations:** 1 Radiology, Northwestern University Feinberg School of Medicine, Chicago, IL, USA; 2 Pediatrics, Northwestern University Feinberg School of Medicine, Chicago, IL, USA; 3 Cardiology, Ann & Robert H Lurie Children's Hospital of Chicago, Chicago, IL, USA; 4 Medical Imaging, Ann & Robert H Lurie Children's Hospital of Chicago, Chicago, IL, USA

## Background

Bicuspid aortic valve (BAV) is a congenital abnormality that is often associated with aortic coarctation. Evidence suggests this combination may increase the risk of secondary aortic complications compared to patients with BAV alone.[1] Altered aortic hemodynamics likely induce vascular remodeling[2] and may contribute to these complications. No study has investigated the impact of aortic coarctation on aortic hemodynamics in pediatric and young adult patients with BAV using time-resolved three-dimensional phase contrast (4D flow) MRI. The aim of this study is to utilize 4D flow MRI to compare thoracic aorta flow patterns in pediatric and young adult patients with BAV with and without aortic coarctation or coarctation repair.

## Methods

In accordance with an IRB-approved protocol, 17 patients with BAV (age = 11.9 +/- 5.3 years) who underwent cardiovascular MRI including 4D flow as part of follow-up care were included. Seven patients had aortic coarctation including 5 patients with coarctation repairs. Images were processed using in-house software for noise reduction, anti-aliasing, and eddy current correction. Flow visualization and quantification were performed in EnSight (CEI, Apex, NC). Aortic root z-scores were calculated from MRI measurements and height and weight at time of scan in EchoIMS (Merge, Chicago, IL). Flow jet pattern and helicity were assessed in the ascending aorta, aortic arch, and descending aorta. Flow jet pattern was determined by the region of peak velocity in systole corresponding to anatomically designated quadrants of planes placed perpendicular to flow in each region of interest (ROI). Helicity was graded on a scale of 0-5 based on the rotation angle within each ROI. Helicity was compared using a Wilcoxon rank-sum test. All other data were compared using a Student's t-test.

## Results

**Figure 1:**
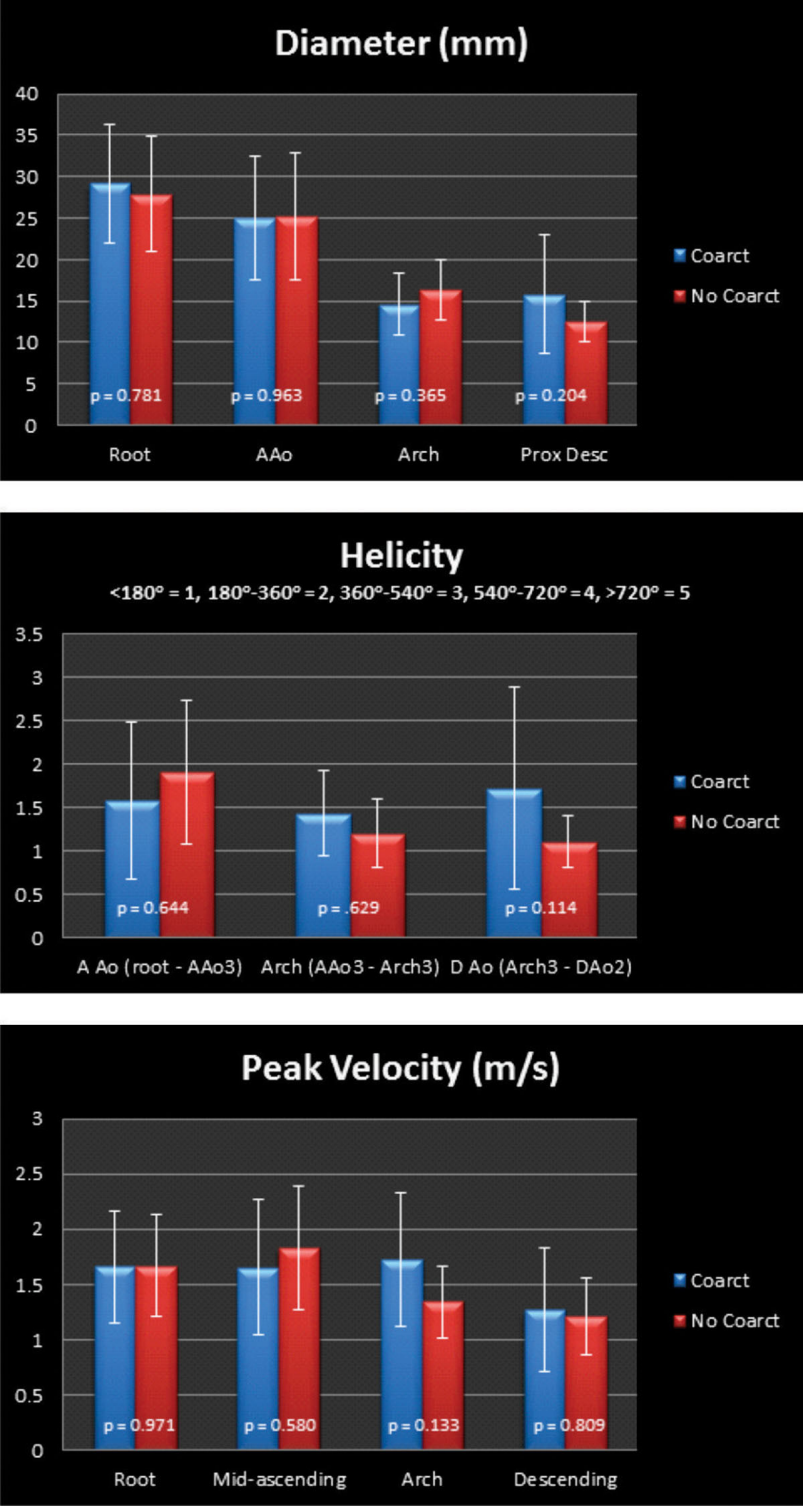
Comparison between Coarctation and No Coarctation groups.

**Figure 2:**
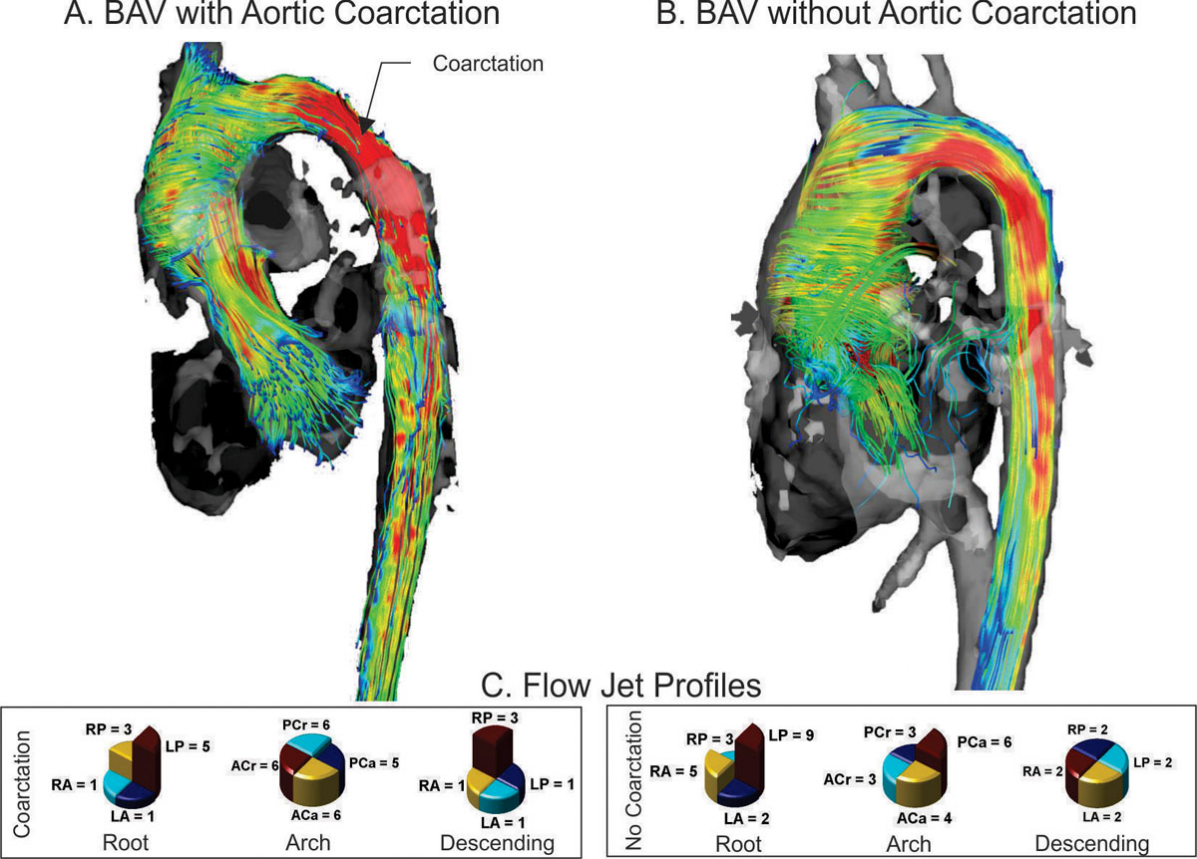
Velocity streamline representation of flow and flow jet profiles. A. Bicuspid aortic valve (BAV) with aortic coarctation. Note the presence of a high velocity flow jet at the site of coarctation and right-handed helical flow in the descending aorta. B. BAV without aortic coarctation. Note lower velocity throughout the arch and descending aorta with nearly laminar flow in the descending aorta. There are right-handed helix formations in the ascending aorta in both cases. C. Flow jet profiles along the aorta in BAV with and without aortic coarctation. There is eccentricity focused posterolaterally in the ascending aorta in both cohorts. There is dramatically increased eccentricity focused posterolaterally in the coarctation cohort. (Quandrants: Left Anterior - LA, Right Anterior - RA, Left Posterior - LP, Right Posterior - RP, Anterior-Cranial - ACr, Posterior-Cranial - PCr, Anterior-Caudal - ACa, Posterior-Caudal - PCa)

There was no significant difference in the age (13.2 +/- 6.5 years vs. 11.0 +/- 4.1 years, p = 0.433) or the root z-scores (2.8 +/- 1.4 vs. 2.9 +/- 2.0, p = .983) between the coarctation (n=7) and non-coarctation (n=10) groups. There was a trend toward higher arch velocities, increased helicity in the descending aorta, smaller arch diameter, and larger descending aorta diameter in the coarctation group, although no difference was statistically significant (Figure 1). There was a high degree of helicity in the ascending aorta in both groups. The coarctation group had more eccentric flow in the descending aorta (Figure 2).

## Conclusions

In pediatric and young adult patients with BAV, aortic coarctation or coarctation repair is associated with a trend towards increased flow derangement relative to the non-coarctation group, but the power to detect a significant difference was limited by cohort size. Future work should focus on correlating outcomes such as aneurysm formation and re-operation with flow characteristics in this population.

## Funding

Grant support: NIH R01HL115828, NUCATS Dixon Award
